# Thiophene–Sulfone-Based D-A Conjugated Porous Polymers: Acceptor Regulation for Efficient Blue Light-Driven Selective Aerobic Oxidation of Sulfides and Amines

**DOI:** 10.3390/molecules31071065

**Published:** 2026-03-24

**Authors:** Ruiyao Li, Fei Zhao, Qun Li, Shuai Feng, Chang-An Wang, Yinfeng Han, Xueli Cheng, Jinsheng Zhao

**Affiliations:** 1 College of Chemistry and Chemical Engineering, Taishan University, Taian 271000, China; 2 College of Chemistry and Chemical Engineering, Qufu Normal University, Qufu 273165, China; 3 College of Chemistry and Chemical Engineering, Liaocheng University, Liaocheng 252059, China

**Keywords:** pyrene, conjugated microporous polymer, donor–acceptor, photocatalytic oxidation, amines

## Abstract

Donor–acceptor (D-A)-type conjugated porous polymers (CPPs) have emerged as highly competitive photocatalysts for aerobic oxidation reactions. Herein, we rationally design and synthesize a series of D-A structured photocatalysts by employing dibenzothiophene-S, S-dioxide (BTDO) as the acceptor unit, and 4,8-bis(thiophen-2-yl) benzo [1,2-b:4,5-b’] dithiophene (DBD) and pyrene (Py) as the donor units. The effects of acceptor content on the optoelectronic and photocatalytic properties are systematically investigated. With the gradual increase in BTDO proportion and the decrease in pyrene content, the photocatalysts exhibit gradually narrowed band gaps, significantly promoted charge separation efficiency, and broadened visible light absorption range. Among the five as-prepared photocatalysts, DBD-T displays superior catalytic performance toward blue light-driven aerobic oxidation. Under mild conditions, benzyl sulfide and benzyl amine are selectively converted into benzyl sulfoxide and benzyl imine with a high conversion efficiency up to 96%. Moreover, DBD-T shows good universality toward a wide range of substrates, together with excellent recyclability and long-term stability. This work demonstrates that enhancing the electron-withdrawing capability of the acceptor unit represents a feasible and effective strategy to boost the photocatalytic performance of D-A-type conjugated polymers.

## 1. Introduction

Oxidation, as a fundamental chemical transformation, plays a vital role in industrial production and daily life [1]. But conventional oxidation methods usually require the use of oxidizing agents and produce a large number of by-products, which may contain toxic heavy metals or be irritating [2]. Therefore, aerobic oxidation using molecular oxygen (O_2_) as a green oxidant is an ideal strategy to solve environmental problems and increase atomic economy [3,4]. However, conventional thermal-driven aerobic oxidation usually demands high temperatures, leading to considerable energy consumption and poor functional group tolerance [1,5]. In recent years, organic synthesis driven by solar energy has received increasing attention due to its environmental friendliness and mild conditions [5]. To date, various photocatalysts including organic dyes [6,7] and metal complexes [8] have been used for aerobic oxidation. These photocatalysts have high catalytic activity due to their homogeneous catalytic properties, but their structural instability and difficulties in recycling have limited their wide application. At the same time, heterogeneous photocatalysts consisting of pure organic compounds have emerged, which have the advantages of non-toxicity, low synthesis cost, structural diversity, wide range of light absorption and satisfactory recyclability [5]. For example, graphitic carbon nitride (g-C_3_N_4_) [9,10,11], crystalline covalent organic frameworks (COFs) [12,13,14,15], covalent triazine frameworks (CTFs) [16,17] and conjugated porous polymers (CPPs) [18,19] have been developed as multiphase visible light photocatalysts.

High-performance heterogeneous photocatalysts typically require strong visible light absorption, efficient charge separation, and fast carrier migration [6]. The construction of donor–acceptor (D-A) conjugated backbones has been established as one of the most powerful strategies to promote intramolecular charge transfer and suppress electron–hole recombination, thereby boosting photocatalytic activity [20,21,22,23]. Previous studies on photocatalytic aerobic oxidation were based on donor units such as pyrene [24], triazine [17], truxene [25], benzotrithiophene [26], etc. Wu et al. prepared two vinyl-bridged D-A porous organic polymers (TpTc-POP and TbTc-POP) from electron-rich triaromatic amines and electron-deficient polycyclic alkynes via Knoevenagel condensation, which exhibited excellent visible light photocatalytic activity for aerobic oxidative coupling of amines to imines [6]. Luo et al. developed a series of D-A POPs featuring a carbazole–triazine-based architecture. These materials demonstrated high efficiency in aerobic oxidation reactions, including sulfide oxidation, amine–oxide coupling, and the Mannich reaction [1]. Pyrene consists of four fused benzene rings, and the planar and highly conjugated structure of pyrene gives it a highly rigid backbone to build porous materials [21,24]. Dong et al. assembled two pyrene-based conjugated microporous polymer (CMPs) (Py-DTDO-2 and Py-DTDO-4) using pyrene and dithiophene (3,2-b:2′,3′-d) thiophene 4,4-dioxide (DTDO) as the donor and acceptor. O_2_ was used as the oxidant, and the amine was rapidly oxidized to form imine on both catalysts under red light irradiation [27]. Although previous CPP-based photocatalysts have achieved notable results, it remains critically important to explore more D-A-type structures for photocatalytic aerobic oxidation and to systematically clarify their structure/performance relationships.

As a rigid planar unit with strong electron-withdrawing ability, dibenzothiophene-S, S-dioxide (BTDO) has been widely used to construct higher performance conjugated polymers [20,21,28]. But it has been rarely applied in the field of photocatalytic oxidation, especially in the fields of photocatalytic aerobic oxidation of sulfides and amines. Kumar et al. employed BTDO as an acceptor and combined it with the strong electron donor pyrrolo[3,2-b]pyrroles via multicomponent reactions to prepare CMPs for the photocatalytic degradation of RhB. Under UV-Vis irradiation, the photogenerated electrons produced on BTDO reduce O_2_ to $O_{2}^{\cdot -}$, which degrades 97.28% of RhB within 3 h [29]. Han et al. synthesized DPB-BTDO via Suzuki–Miyaura polymerization of BTDO and the highly planar 1,4-diphenylbutadiyne (DPB) donor. With suitable band edge positions, it efficiently produces H_2_O_2_ (4.79 mmol·h^−1^·g^−1^) from pure water under visible light without sacrificial agents. Here, the $O_{2}^{\cdot -}$ generated from the reduction of O_2_ by BTDO-based photogenerated electrons acts as the key intermediate [30]. These studies demonstrate that BTDO has potential to serve as an efficient electron acceptor in D-A photocatalysts for versatile photocatalytic reactions. 4,8-bis(thiophen-2-yl) benzo [1,2-b:4,5-b’] dithiophene (DBD) contains a thiophene ring and exhibits enhanced light absorption owing to its stabilized and extended π-conjugated structure [31,32]. Li et al. reported CPPs with light-trapping chromophores and transition metal-binding bipyridine sites. The DBD-containing CPPs delivered the highest H_2_ production rate of 33 μmol/h, demonstrating that copolymerizing strong donors with weak acceptors is an effective strategy for high-performance photocatalysts [31]. Although BTDO or DBD-containing polymers have been explored for hydrogen generation, the rational combination of DBD and BTDO into a D-A CPP for light-driven aerobic oxidation of amines and sulfides represents an unprecedented approach. To date, this specific D-A pairing has not been reported for aerobic oxidation reactions.

**Scheme 1:**
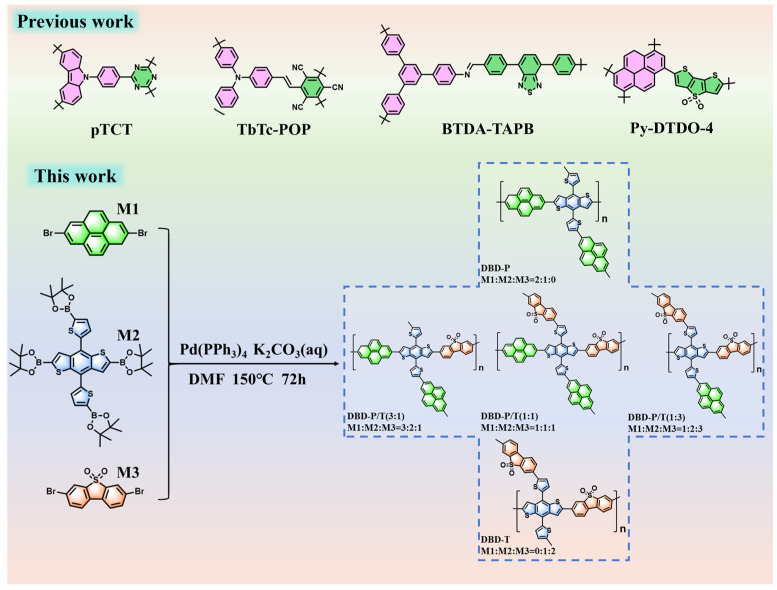
Several typical donor and acceptor combinations for photocatalytic aerobic oxidation reactions in previous works (pTCT [1], TbTc-POP [6], BTDA-TAPB [15], and Py-DTDO-4 [27]). Synthetic routes to polymers with different acceptor ratios in this work. (The ratios (e.g., 3:2:1, etc.) denote the molar feed ratios).

In order to gain insight into the effects of chemical structure and monomer composition on photocatalytic activity, a series of CPPs with two different donor units (M1: Pyrene and M2: DBD) and one acceptor unit (M3: BTDO) were fabricated (Scheme 1). The effects of chemical structure, especially monomer composition, on the photocatalytic activity of D-A copolymers were systematically investigated by adjusting the feed ratio of donor to acceptor. Here, the polymers were synthesized via a Pd-catalyzed Suzuki–Miyaura polymerization to construct five photocatalysts for CPPs, named DBD-P, DBD-P/T (3:1) DBD-P/T (1:1) DBD-P/T (1:3) and DBD-T. The structures and photocatalytic oxidation performances of five conjugated porous polymers (CPPs) were systematically investigated. All CPPs generate reactive oxygen species (ROS) and enable photocatalytic oxidative amine coupling and thioether oxidation under blue light irradiation in oxygen-saturated solvents. Notably, DBD-T exhibits superior photocatalytic oxidation activity, which is attributed to the fully conjugated multi-directional linkage provided by DBD units and the high coplanarity of BTDO that suppresses electron–hole recombination and accelerates charge transport.

## 2. Results and Discussion

### 2.1. Morphology and Structure

**Figure 1:**
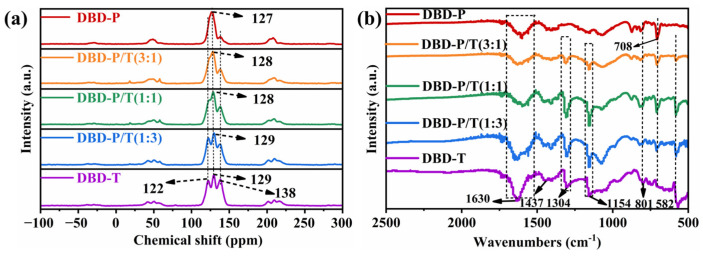
(**a**) Solid-state ^13^C CP-MAS NMR spectra and (**b**) FTIR spectra of the polymers.

Five polymers in the state of insoluble solid powder with different sulfone contents were synthesized using Pd-catalyzed Suzuki–Miyaura polymerization, and the apparent color of the polymers varied from green to orange to purplish-red as the sulfone content was increased [33]. The chemical structure of the copolymers was characterized by Fourier transform infrared spectroscopy (FTIR) and the solid-state ^13^C CP-MAS NMR. The carbon resonances of the five polymers were then determined by the solid-state ^13^C CP-MAS NMR. As can be seen in Figure 1a, the five polymers exhibit the same chemical shift at 138 ppm, and this signal peak corresponds to the carbon atoms at the S-atom attachment sites in the sulfone and thiophene groups in the polymers, and the area of the carbon atom peaks was gradually enhanced with the increase in the BTDO content from DBD-P to DBD-T [20,34,35]. The peak at 127 (128 or 129) ppm signals aromatic carbon [35]. Subsequently, the different functional groups in DBD-P and DBD-T were clearly shown by Fourier transform infrared (FTIR) spectroscopy (Figure 1b). The vibrational signals are located at 1154 and 1304 cm^−1^ for the sulfone, which is an asymmetric stretching absorption typical of O=S=O [32]. Characteristic signals of the aromatic skeleton can be observed at 1437 and 1630 cm^−1^, and 1507 cm^−1^ and 1412 cm^−1^ show the presence of aromatic pyrene [18,36]. In Figure 1b, the other three polymer peaks are in approximately the same position, but the peak intensities are slightly different, which may be caused by building polymers with the same monomers in different proportions. All these peaks confirm the successful formation of the polymer.

**Figure 2:**
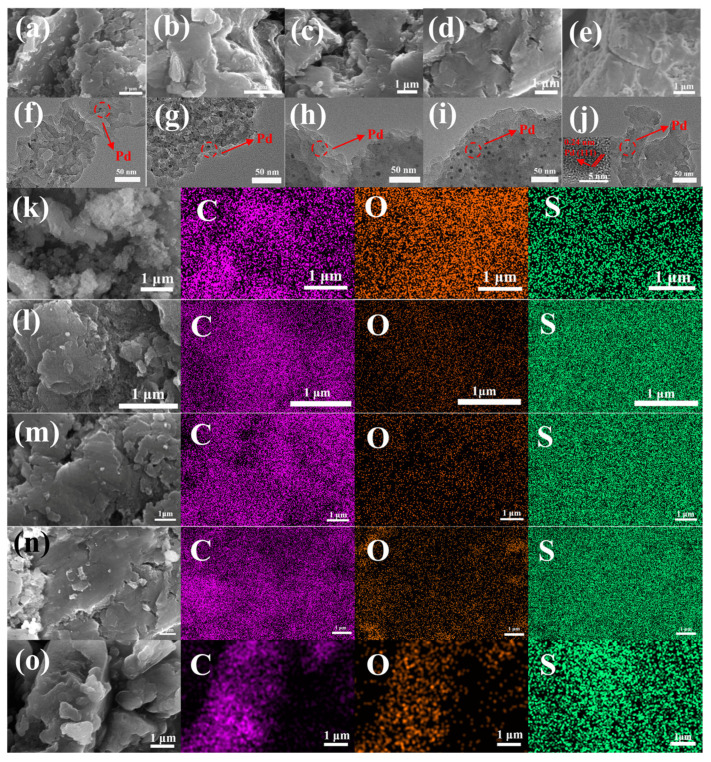
SEM images for DBD-P (**a**), DBD-P/T (3:1) (**b**), DBD-P/T (1:1) (**c**), DBD-P/T (1:3) (**d**), DBD-T (**e**); TEM images for DBD-P (**f**), DBD-P/T (3:1) (**g**), DBD-P/T (1:1) (**h**), DBD-P/T (1:3) (**i**), DBD-T (**j**); element mapping images for DBD-P (**k**), DBD-P/T (3:1) (**l**), DBD-P/T (1:1) (**m**), DBD-P/T (1:3) (**n**), DBD-T (**o**).

The morphology of these photocatalysts was investigated using scanning electron microscopy (SEM) and transmission electron microscopy (TEM). The SEM images (Figure 2a–e) showed that all the polymers exhibited a block structure consisting of thin sheets. Moreover, the lamellar structure could be observed in the TEM images of these polymers, which was due to the lamellar structure of the bulk polymer stripped out by the sonication process. In the high-resolution TEM (Figure 2f,g), there is a small amount of residual palladium particles in the polymers, which might function as the residual palladium catalyst during the synthesis of the polymers [18]. The residual Pd contents measured by inductively coupled plasma mass spectrometry (ICP-MS) are 0.60 wt% (DBD-P), 0.54 wt% (DBD-P/T(3:1)), 0.50 wt% (DBD-P/T(1:1)), 0.63 wt% (DBD-P/T(1:3)), and 0.57 wt% (DBD-T). The d spacing obtained from the lattice fringes (inset of Figure 2f) is 0.25 nm, which could be the (111) plane of the nano-Pd. Elemental mapping images (Figure 2k–o) of the five polymers are given, with elements such as C, S, and O dispersed in the polymers, which is consistent with the actual composition of the polymers. To check the thermal stability of the polymers, TGA (Figure S2) was performed and the TGA results indicated that the initial decomposition temperatures of the polymers were 448 °C, 428 °C, 566 °C, 514 °C, and 334 °C. The aggregation state of the five photocatalysts was tested by powder X-ray diffraction spectrum (PXRD). None of the polymers showed sharp diffraction peaks in Figure S3, indicating that these polymers have an amorphous structure.

In addition, the permanent porosity of the five catalysts was evaluated at 77 K using N_2_ adsorption and desorption isotherms. The adsorption isotherms of N_2_ for all five polymers showed type III isotherms (Figure S4), suggesting that meso- and macroporous structures may have been formed between the sheet photocatalysts by stacking. The Brunauer–Emmett–Teller (BET) surface areas (SBET) of DBD-P (a), DBD-P/T (3:1) (b), DBD-P/T (1:1) (c), DBD-P/T (1:3) (d) and DBD-T (e) were 30.55 m^2^/g, 86.65 m^2^/g, 11.97 m^2^/g, 15.46 m^2^/g and 10.29 m^2^/g, which shows that there is no obvious pattern in the SBET values of the five catalysts, indicating that the specific surface area of the catalysts is not the main factor affecting the photocatalytic activity. The inset in Figure S4 shows the pore size distribution of the polymers with average mesoporous particle sizes of 27.2738 nm, 11.64223 nm, 8.0242 nm, 6.5794 nm, and 7.4952 nm for DBD-P (a), DBD-P/T (3:1) (b), DBD-P/T (1:1) (c), DBD-P/T (1:3) (d) and DBD-T (e), respectively.

**Figure 3:**
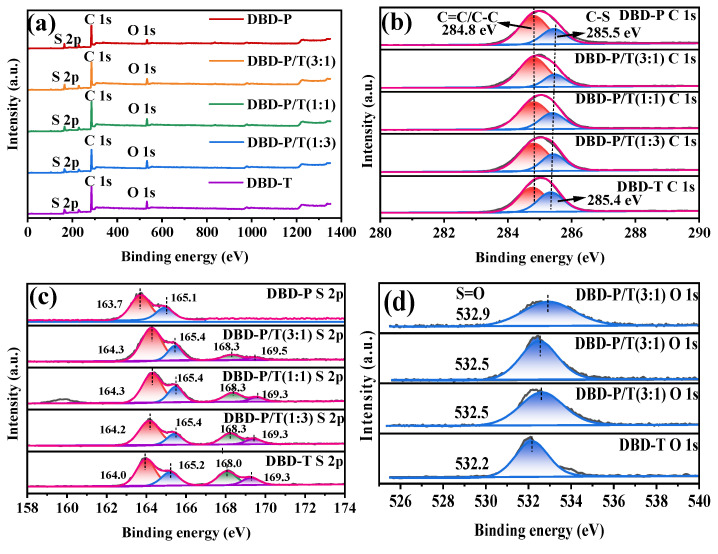
(**a**) XPS survey spectrum; (**b**) C 1s; (**c**) S 2p; and (**d**) O 1s spectra of polymers.

To further determine the chemical composition of the polymers, XPS analysis was also performed. In the total XPS measurement spectra (Figure 3a), it can be seen that the polymers all contain the three elements of sulfur (S), carbon (C) and oxygen (O), with the O element in DBD-P being derived from H_2_O [28]. Their elemental species are further described in the single-element XPS spectra. In Figure 3b, we can see two peaks located at 284.8 and 285.5 eV (or 285.4 eV), which correspond to the C-C=C or C-C in the aromatic ring, and C-S bonds in the thiophene or BTDO, respectively, with a displacement of the peaks for carbon, which is related to the different contents of BTDO [37]. For the S 2p spectra (Figure 3c), in the polymer DBD-P, the peaks with binding energies at 163.7 eV and 165.1 eV belong to S 2p_3/2_ and S 2p_1/2_ of the S atoms in the C-S-C unit in the thiophene unit. In the polymer DBD-T, the S 2p_3/2_ peak 164.0 eV and the S 2p_1/2_ peak 165.2 eV are due to the carbon bonding in the thiophene units, and the 168.0 S 2p_3/2_ peak eV and 169.3 S 2p_1/2_ eV are due to the O=S=O in the BTDO units [38,39,40]. For ternary polymers, the XPS spectra are similar to DBD-T. In addition, the area of the O=S=O peaks gradually increased with the increase in the ratio of BTDO units, and the binding energy continuously increases from 169.5 eV to 169.3 eV. This may be attributed to the strong electro-absorption ability of BTDO to drive the charge mobility energy to higher domains. For the O 1s spectrum (Figure 3d), the peak with a binding energy of 532.9 eV (532.5 eV or 532.2 eV) can be attributed to the S=O unit in BTDO [18]. The above results further confirm the successful preparation of the polymer. The XPS data shown above indicate that these polymers have been successfully prepared.

### 2.2. Spectral Response and Energy Band Analysis

**Figure 4:**
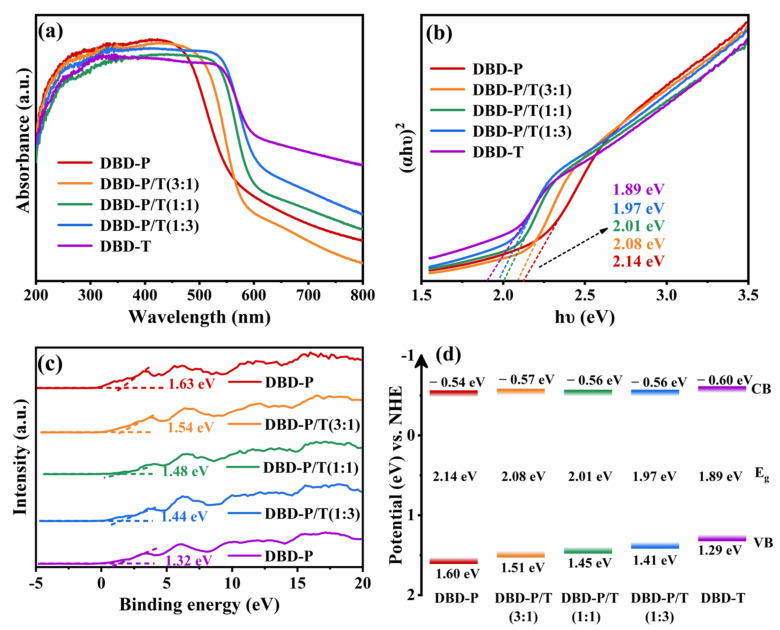
(**a**) UV-Vis DRS; (**b**) *Tauc* plots; (**c**) XPS valence band spectra; and (**d**) band structure diagrams of the five polymers.

The range of light absorption plays a decisive role in the photocatalysis of semiconductors. The light absorption properties of five polymers were then evaluated using UV-Vis diffuse reflectance spectroscopy (DRS). Apparently, the absorption edges of the five polymers are redshifted in sequence with increasing BTDO content, and DBD-T exhibits the broadest absorption region. The broad UV-Vis absorption (Figure 4a) indicates a higher degree of conjugation, which allows higher electron delocalization, which facilitates the photocatalytic reaction under visible light [41]. The *Tauc* diagram (Figure 4b) was plotted based on the Kubelka–Munk formula [42] (Formula (1)) and the UV diagram, from which the optical band gaps of the five polymers are 2.14 eV (DBD-P), 2.08 eV (DBD-P/T (3:1)), 2.01 eV (DBD-P/T (1:1)), 1.97 eV (DBD-P/T (1:3)) and 1.89 eV (DBD-T).

(1) $$\alpha h\nu ={A(h\nu -E_{g})}^{1/2}$$

Furthermore, the valence band maximum (*E*_VBM_) values of the polymers were obtained by valence band XPS (VB-XPS) (Figure 4c) as 1.63 eV, 1.54 eV, 1.48 eV, 1.44 eV and 1.32 eV. The valence band maxima were converted using Formula 2 [38]. The valence band (VB) potentials (*E*_VB_) potentials for DBD-P, DBD-P/T(3:1), DBD-P/T(1:1), DBD-P/T(1:3) and DBD-T are 1.60, 1.51, 1.45, 1.41 and 1.29 eV vs. NHE, respectively. The conduction band (CB) potentials (*E*_CB_) values of the five polymers were calculated according to Equation 3 (Figure 4d) and the calculated *E*_CB_ values were −0.61, −0.65, −0.48, −0.67, and −0.50 eV, respectively.

(2) $$E_{VB}\left(vs.\;NHE\right)=\varphi +E_{VBM}-4.5\;\mathrm{eV}$$

(3) $$E_{VB}=E_{CB}+E_{g}$$

The Mott–Schottky diagrams were also analyzed at different frequencies of 500, 1000 and 1500 Hz (Figure S5). The positive slopes in the Mott–Schottky plots of the five polymers indicate their apparent n-type semiconductor characteristics. The flat band potentials (*E*_fb_) were calculated from the x-axis intersection values. For the five polymers, these values were −0.75, −0.78, −0.77, −0.77, and −0.81 V (vs. Ag/AgCl). Values of −0.54, −0.57, −0.56, −0.56, and −0.60 eV (vs. NHE) were obtained by transformation [43]. The LUMO (lowest unoccupied molecular orbital) levels of the five polymers were more negative than the LUMO levels of ${O_{2}/O}_{2}^{\cdot -}$ (−0.33 eV vs. NHE). Thus, they both activate oxygen sufficiently to turn it into ROS for aerobic oxidation. In addition, the VB calculated by VB-XPS for the polymers corresponds to the *E*_fb_ estimated from the Mott–Schottky diagram.

### 2.3. Generation and Separation of Charge Carriers

**Figure 5:**
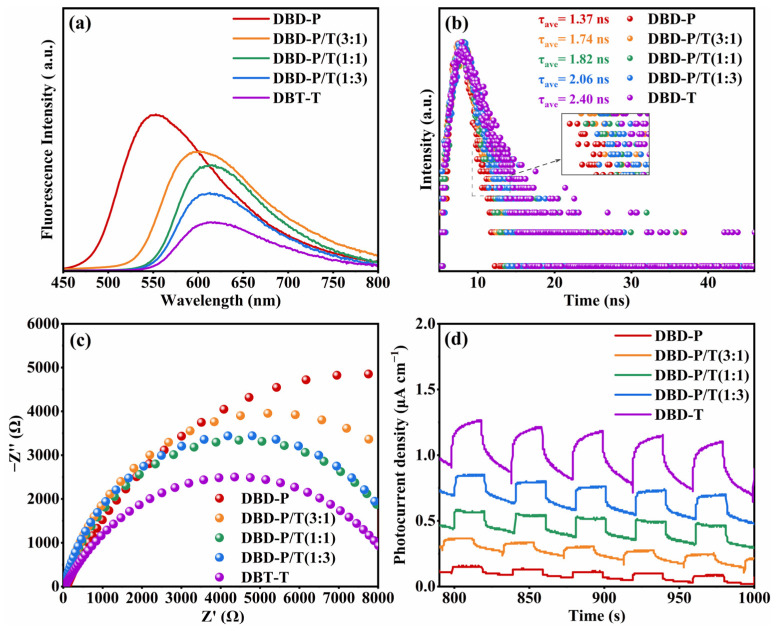
(**a**) Photoluminescent spectra of the polymers; (**b**) fluorescence decay spectra of the polymers; (**c**) fitted Nyquist impedance plots; and (**d**) transient photocurrent of polymers measured in 0.5 M Na_2_SO_4_ solution.

The production and separation of polymer photogenerated carriers are crucial for aerobic oxidation reactions. From the photoluminescence (PL) spectra (Figure 5a), it can be seen that the PL emission peaks of DBD-P/T (1:1), DBD-P/T (1:3), and DBD-T show a significant redshift compared to those of DBD-P, and DBD-P/T (3:1), a phenomenon that further indicates a narrowing of the bandgap with the increase in BTDO content. DBD-T has the lowest PL emission intensity, which implies that more light-induced electrons are involved in the photocatalytic reaction rather than complexing with photogenerated holes via fluorescent radiation, which is favorable for improving the photocatalytic activity [43,44]. The PL curve of DBD-P is significantly different from that of the other four polymers, owing to its poor photoinduced charge separation and the faster recombination of electrons and holes, which is consistent with the fluorescence lifetime analysis results in Figure 5b. Subsequently, the excited-state lifetimes of the five polymers were measured by time-resolved fluorescence decay spectroscopy using a 405 nm laser excitation source (Figure 5b). As shown in Table S1, the *τ*_*ave*_ values were 1.37 ns, 1.74 ns, 1.82 ns, 2.06 ns and 2.40 ns. Obviously, the average fluorescence lifetime of DBD-T was significantly longer than that of the other four photocatalysts, which suggests that DBD-T exhibits a slower rate of electron–hole complexation, which is conducive to the enhancement of photocatalytic activity [34].

In order to further investigate the electron transfer between the polymers, electrochemical impedance spectroscopy (EIS) and time-transient photocurrent response tests were performed on five different polymers. Figure 5c shows the fitted Nyquist plot of the five samples, and the inset also gives the equivalent circuit diagram of the CPPs. In the circuit diagram, the charge transfer resistance (R_p_), which represents the charge transfer resistance between the sample and the electrolyte, is influenced by the charge separation efficiency and the physical interfacial conditions. According to the equivalent circuit fitting (detailed parameters listed in Table S2), the resistances following the order of 15.12 KΩ (DBD-P), 10.53 KΩ (DBD-P/T (3:1)), 9.03 KΩ (DBD-P/T (1:1)), 8.94 KΩ (DBD-P/T (1:3)), and 8.67 KΩ (DBD-T). Due to the higher ratios of acceptor, DBD-T exhibits the smallest R_p_ among the five polymers, indicating that DBD-T has the lowest charge transfer resistance at the interface. Previous data have shown that a smaller charge transfer resistance has a more efficient carrier mobility, which is more conducive to the transport of photogenerated charges and improves the photocatalytic activity [45,46]. Ultimately, under the combined effects of a greater number of photogenerated electrons participating in the reaction, a lower carrier recombination rate, and a faster carrier migration rate, a higher photocurrent density is achieved. As can be seen from the plots (Figure 5d), the DBD-T photocatalyst has the highest photocurrent density, indicating that DBD-T can generate more photoinduced charges during the photocatalytic process, which is an important contribution to the photocatalytic oxidation reaction.

### 2.4. Photocatalytic Performance

**Figure 6:**
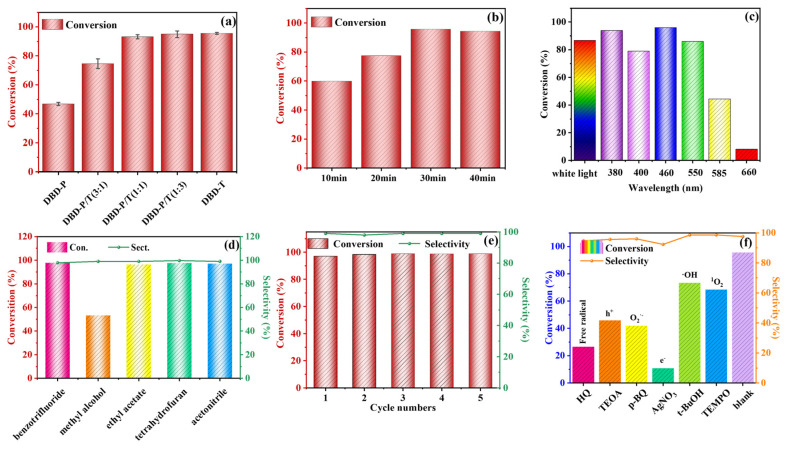
(**a**) Photocatalytic oxidation of benzylamine by different catalysts. (**b**) Selective oxidation of benzylamine by DBD-T at different reaction times. Reaction conditions: DBD-T (2 mg), benzylamine (0.3 mmol), CH_3_CN (1 mL), O_2,_ r.t, blue LEDs. (**c**) Role of light source wavelength on the photocatalytic selective oxidation of benzylamine by DBD-T. Reaction conditions: DBD-T (2 mg), benzylamine (0.3 mmol), CH_3_CN (1 mL), 0.5 h, O_2,_ r.t. (**d**) Selective oxidation of benzylamine by DBD-T in different solvents. Reaction conditions: DBD-T (2 mg), benzylamine (0.3 mmol), solvent (1 mL), O_2,_ r.t, blue LEDs. (**e**) Recycling for the selective oxidation of benzylamine. Reaction conditions: benzylamine (0.3 mmol), DBD-T (2 mg), CH_3_CN (1 mL), blue LEDs, O_2_, 0.5 h, r.t. (**f**) DBD-T catalyzed burst experiment for the oxidation of benzylamine. Reaction conditions: benzylamine (0.3 mmol), DBD-T (2 mg), solvent (1 mL), blue LEDs, O_2_, 0.5 h, r.t, 0.5 equivalent bursting agent.

Firstly, the photocatalytic performance of five polymers for the selective oxidation of amines to imines was investigated using benzylamine as a model substrate. As clearly shown in Figure 6a, the reactivities of DBD-P and DBD-P/T (3:1) driven by blue light were significantly lower than those of the other three polymers. Notably, there is no positive correlation between the conversion rate and the residual amount of Pd. In particular, DBD-P/T (1:1) and DBD-T with lower Pd residues exhibit higher photocatalytic conversion rates. Therefore, the large difference may be due to the increased content of BTDO, which makes the conjugated backbone larger and can absorb more light in the blue light range. Subsequently, DBD-T was selected as the optimal catalyst for the effect of time on catalytic performance. Finally, 30 min was selected as the optimal reaction time (Figure 6b). Secondly, the effect of the dosage of DBD-T on its photocatalytic aerobic oxidation ability was evaluated. Catalytic oxidation experiments were carried out on benzylamine with 2, 3, 4 and 5 mg of catalyst selected sequentially, and the results are shown in Table S3, which show that the amine conversion and selectivity increased slightly with the increase in catalyst dosage. Therefore, in order to protect the activity of the catalyst, the amount of catalyst was set at 2 mg in the subsequent experiments. In addition, the effect of LEDs of different wavelengths on the photocatalytic activity was also investigated using benzylamine as the substrate. As shown in Figure 6c, the corresponding yields basically coincided with the UV-Vis absorption spectra of the polymers. The photocatalysts maintained high activity under white light or 550 nm, especially good selectivity for the aerobic oxidation of benzylamine under both blue and violet light, and therefore, blue LEDs were selected as the experimental light source for the subsequent experiments.

The effect of different solvents on the reaction is usually considered as an important factor. Therefore, the photocatalytic activity of DBD-T was further examined in different organic solvents including benzotrifluoride, methanol, ethyl acetate, tetrahydrofuran and acetonitrile. As shown in Figure 6d, the solvent used at the highest conversion (97%) was acetonitrile (ACN), which was therefore considered as the most ideal solvent for the amine conversion experiments. In the oxidation of sulfoxide (Table S4), the use of methanol (MeOH) as the solvent resulted in a high conversion (96%) and selectivity (99%) for the selective oxidation of p-methylphenyl sulfide. This is attributed to the fact that aerobic oxidation of sulfoxide typically involves proton exchange, and methanol demonstrates a strong capacity for proton donation. The desirable catalytic performance is on par with that of our previously reported D-A conjugated polymer systems for photocatalytic aerobic oxidation, where truxene [25], benzotrithiophene [26], and triazine [47] were used as donor units.

Multiphase catalysts have attracted much attention due to their satisfactory recoverability, and therefore, cycling experiments are used to test the recoverability of polymers. The catalyst after the aerobic oxidation reaction needs to be first filtered under reduced pressure and washed with methanol and tetrahydrofuran, and the cleaned catalyst is dried in a vacuum oven and used for the next cycle test. After five cycles, the conversion and selectivity of the catalyst did not decrease significantly (Figure 6e), and the conversion was always higher than 95% and the selectivity was higher than 98%.

At the end of the optimization experiments, the versatility of DBD-T was further verified by selecting various amine compounds under optimal conditions. We chose 11 amine compounds (Table 1) as substrates and explored the effects of substituent effects and spatial site resistance on the photocatalytic oxidation activity of DBD-T. Due to the stronger electron-withdrawing nature of -F, -Cl, and -Br, the electron density of the benzylamine group decreased, making it more difficult to generate amine radical cations through oxidation by photogenerated holes. As a result, the reaction rate slowed down, leading to a substantially lower benzylamine conversion of para-halogen substituents (Table 1, entries 2–4) within the same reaction period. Therefore, extending the reaction time to 0.9 h led to a notable enhancement in their conversion rates (Table S5, entries 2–4). In contrast, substrates with strongly electron donating para-substituents exhibited high conversion efficiencies in 0.5 h (Table 1, entries 5, 7, and 9). When the substituent was shifted from the para- to the ortho-position, both the conversion of substrates (Table 1, entries 6 and 8) with electron-withdrawing or electron-donating were unsatisfactory. Even if the reaction time was extended to 0.9 h, the conversions all failed to exceed 90% because of the relatively large steric hindrance (Table S5, entries 6 and 8). For hetero-substituted primary amines (Table 1, entries 10 and 11), DBD-T gives them satisfactory conversions.

Subsequently, the photocatalytic aerobic oxidation of thioanisole to sulfoxide was applied as another model reaction to assess the photocatalytic activity on other organic transformations (Table 2). The conversion of thioanisole oxidation at the same time was lower than that of benzylamine, due to the higher energy barrier of the former reaction [48]. The aromatic sulfides containing electron-donating substituent groups (Table 2, entry 1 and 5) typically retain higher conversions compared to electron-withdrawing ones (Table 2, entries 2 and 3). Due to the slow reaction kinetics, the conversion of 1-bromo-4-methoxybenzene was as low as 33%. When methyl was replaced by benzyl with stronger steric hindrance (Table 2, entry 4), the corresponding substrate benzyl phenyl ether exhibited a lower conversion (54.75%), but it increased to 75% at the condition of 0.9 h (Table S6, entry 4). This phenomenon further demonstrates the importance of substituent and steric hindrance effects in the reaction.

### 2.5. Reaction Mechanism

In this part, the controlled experiments were used to explore the photocatalytic mechanism of DBD-T (Tables S4 and S7). The aerobic oxidation reaction does not occur in experiments without catalyst, light irradiation and when the O_2_ atmosphere is changed by a N_2_ atmosphere, and the conversion of the substrate is significantly reduced when the O_2_ atmosphere is changed by an air atmosphere, confirming the indispensable presence of light, catalyst, and oxygen for a successful photocatalytic reaction to occur. Therefore, quenching experiments were carried out using a different reactive species scavenger to determine the excavation of critical ROS (Figure 6f). No significant difference in product yield was observed when tert-butyl alcohol and 2,2,6,6-tetramethyl-L-piperidine-N-oxyl (TEMPO) were respectively added to the reaction solution as OH- scavenger and ^1^O_2_ scavenger. However, the conversion and selectivity of the target product were significantly reduced by the addition of p-benzoquinone as scavenging agent under the same reaction conditions. It can be inferred that the chemical formed during the reaction is the central ROS of the photocatalytic system. Meanwhile, the addition of isopropyl alcohol and AgNO_3_ as h^+^ and e^−^ scavengers resulted in negligible conversions, suggesting the involvement of h^+^ and e^−^ in the oxidation process.

**Figure 7:**
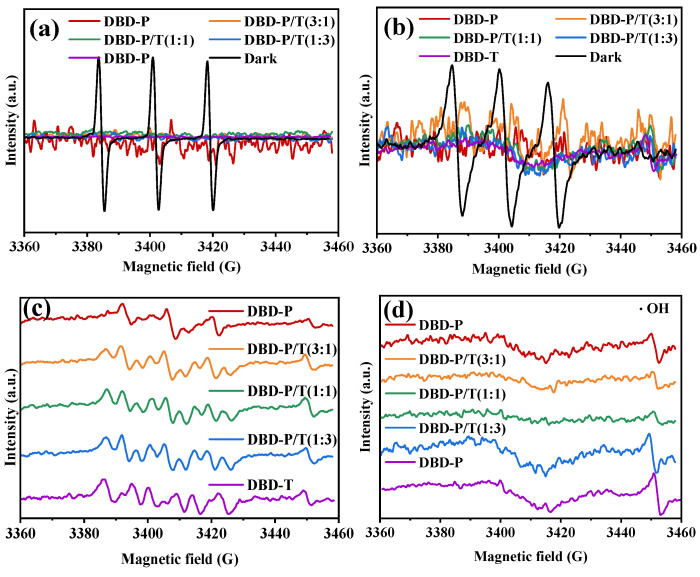
(**a**) EPR spectra of detecting photoexcited electrons in an aqueous solution of polymers; (**b**) EPR spectra of detecting photoexcited holes in an acetonitrile solution of polymers; (**c**) DMPO spin-trapping EPR image of polymers for DMPO-$O_{2}^{\cdot -}$; and (**d**) DMPO-·OH under light irradiation.

Subsequently, electron paramagnetic resonance experiments (EPR) under light conditions were conducted for further probing of possible free radicals generated in the photocatalysts. Firstly, TEMPO was dispersed in an aqueous or acetonitrile solution to act as a trapping agent for electrons or holes. Immediately after, appropriate amounts of catalyst and trapping agent were pipetted and mixed, and the resulting mixture was placed in dark conditions for 30 min before the photocatalyst was filtered out and the supernatant was retained and analyzed. The characteristic triplet peaks of the photocatalyst were observed in both electron and hole trapping experiments, indicating that the TEMPO was able to maintain the original structure at this time. Subsequently, the mixture was placed under light irradiation for 30 min, and the characteristic signals were almost negligible, which could be attributed to the fact that the photogenerated electrons and holes formed by the catalyst under light radiation further combined with TEMPO to form TEMPOH (Figure 7a) and TEMPO^+^ (Figure 7b). At the same time, DMPO in aqueous or methanol solution was used as a trapping agent for ·OH or $O_{2}^{\cdot -}$. None of the five catalysts produced the DMPO-·OH signal due to the fact that their *E*_VB_ potentials (1.54 eV vs. NHE) were lower than those of OH/∙OH (1.99 eV vs. NHE) (Figure 7d). However, all five photocatalysts were able to produce DMPO-$O_{2}^{\cdot -}$ signals, indicating that their *E*_CB_ potentials were negative compared to the redox potential of ${O_{2}/O}_{2}^{\cdot -}$ (−0.33 eV vs. NHE) (Figure 7c), and further verifying the conclusion of the bursting experiments that the $O_{2}^{\cdot -}$ is an indispensable ROS involved in the oxidation reaction.

Based on the above results as well as previously reported results, a reaction mechanism regarding the selective photocatalytic benzylamine coupling and thioether oxidation of DBD-T driven by blue light is proposed. Under irradiation with blue LEDs, electrons within the polymer are transferred from VB to CB, leading to the generation of electron–hole pairs. Subsequently, the holes on the photocatalyst VB induced the generation of benzylamine- or thioalkyl ether-based substrates to form the corresponding amine or thioether cationic radicals. At the same time, photogenerated electrons on the CB oxidize molecular oxygen in the air to the superoxide radical $O_{2}^{\cdot -}$ and the single-linear state oxygen O21. The ROS reacts with benzylamine to form benzylamine intermediates or with thiomethyl ether to form peroxynitrite intermediates, which react with benzylamine and thiophenyl ether, respectively, completing the catalytic cycle to give the desired N-benzyl aniline and peroxynitrite products.

## 3. Materials and Methods

### 3.1. Synthesis of Polymers

These photocatalysts were synthesized by Pd-mediated Suzuki–Miyaura polymerization. Monomer M3 as well as other synthesis processes from the catalyst are in the Supplementary Materials (Section S1.3). DBD-T is used as an example of a representative experimental program: M3 (699.1 μmol, 251.7 mg) and M2 (331.4 μmol, 300.0 mg) were dispersed into a 25 mL flask containing aqueous potassium carbonate (2 M, 12 mL) and N,N-Dimethylformamide (DMF) (30 mL). Subsequently, the reaction flask was evacuated and backfilled three times with nitrogen. Pd(PPh_3_)_4_ (30.0 μmol, 35 mg) was then added to the flask under a N_2_ atmosphere. The mixture was heated and stirred at 120 °C for 72 h under nitrogen as a protective atmosphere. At the end of the reaction, it was filtered through reduced pressure and washed with deionized water, methanol and chloroform. The product was dried under vacuum at 60 °C for 12 h to give a purple-red powder. Product quality: 230 mg; yield: 55.60%.

### 3.2. Standard Method for the Oxidation Reaction

Firstly, the solvent (acetonitrile or methanol) (1 mL), polymer (2 mg) and substrate (0.3 mmol) were sequentially added to a glass-tube reactor (10 mL), which was subsequently sealed and filled with oxygen after three consecutive degassing cycles, and a balloon was used to maintain positive pressure. Next, stirring was carried out for 30 min in the dark using a magnetic stirrer with the aim of achieving an adsorption/desorption equilibrium. Afterward, 24 W blue light-emitting diode (LED) illumination was used for uniform stirring. Once the reaction was finished, the photocatalyst was filtered off after the solvent was used to analyze the conversion of the substrate and the selectivity of the product.

### 3.3. Durability Test for Photocatalysts

The durability of photocatalysts was evaluated through five repeated experiments under standard conditions (benzylamine (0.3 mmol), DBD-T (2 mg), CH_3_CN (1 mL), blue LEDs, O_2_, 0.5 h, r.t). After the reaction, the photocatalyst was recovered by centrifugation, washed with MeOH for removing substrates and products, and dried in a vacuum oven at 60 °C for 12 h. The dried photocatalyst was directly used for subsequent operations without any additional treatment.

## 4. Conclusions

In summary, we constructed a series of D-A-type conjugated porous polymers with different chemical compositions by varying the feed ratios of electron donors and acceptors, and further explored their applications in the photocatalytic aerobic oxidation of amines and sulfides. The experimental results showed that the photoelectric properties and catalytic activities of the polymers showed a certain pattern with the gradual increase in the acceptor content in the catalyst. Compared with DBD-P, DBD-T exhibited enhanced photocatalytic activity, which can be attributed to the increase in the content of the electron acceptor, which enhances the charge separation and transfer ability along the polymer backbone, widens the light absorption range and inhibits charge recombination, on the one hand, and increases the reactive active sites (S elements), on the other hand. The present study shows that the integration of the electron acceptor into the conjugated skeleton by changing the electron acceptor-to-donor feed ratio is promising for the rational design of a variety of D-A structured CPPs with enhanced photocatalytic aerobic oxidation performance.

## Figures and Tables

**Table 1:** The blue light-mediated oxidation of substrates on DBD-T with O_2_ to the corresponding products ^a^.

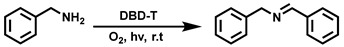					
Entry	Substrate	Product	t [h]	Conv. (%) ^b^	Sel (%) ^b^
1	** 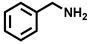 **	** 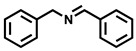 **	0.5	96	98
2	** 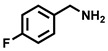 **	** 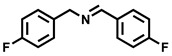 **	0.5	86	>99
3	** 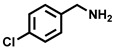 **	** 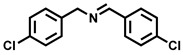 **	0.5	55	>99
4	** 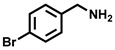 **	** 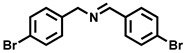 **	0.5	55	>99
5	** 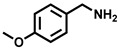 **	** 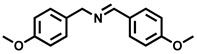 **	0.5	99	98.77
6	** 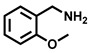 **	** 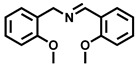 **	0.5	67	>99
7	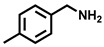	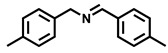	0.5	93	>99
8	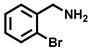	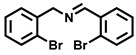	0.6	59	99.00
9	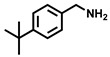	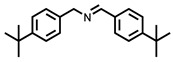	0.6	69	>99
10	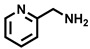	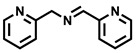	0.6	81	>99
11	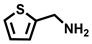	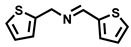	0.6	61	>99

^a^ Reaction conditions: substrate (0.3 mmol), photocatalyst (2.0 mg), ACN (1.0 mL), blue LEDs, r.t, O_2_ (1 atm), 0.5 h. ^b^ Conversion and selectivity of the substrates were determined by ^1^H-NMR.

**Table 2:** The photocatalytic aerobic oxidation of sulfides with oxygen over DBD-T driven by blue light ^a^.

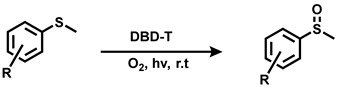					
Entry	Substrate	Product	t [h]	Conv. (%) ^b^	Sel (%) ^b^
1	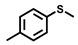	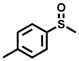	0.5	96	99
2	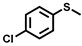	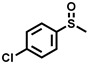	0.5	69	94
3	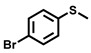	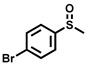	0.5	33	94
4	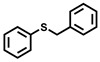	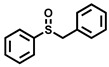	0.7	55	96
5	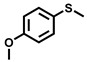	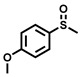	0.7	99	95

^a^ Reaction conditions: substrate (0.3 mmol), photocatalyst (2.0 mg), MeOH (1.0 mL), blue LEDs, r.t, O_2_ (1 atm), 0.5 h. ^b^ Conversion and selectivity of the substrates were determined by ^1^H-NMR.

## Data Availability

The data presented in this study are available on request from the corresponding author.

## Supplementary Materials

The following supporting information can be downloaded at https://www.mdpi.com/article/10.3390/molecules31071065/s1: Figure S1: ^1^H NMR (a) and ^13^C NMR (b) spectrum of 3,7-dibromodibenzothiophene-S,S-dioxide; Figure S2: Thermogravimetric curves of polymers; Figure S3: X-ray diffraction spectra of polymers; Figure S4: Nitrogen isothermal adsorption and desorption curves of DBD-P (a), DBD-P/T (3:1) (b), DBD-P/T (1:1) (c), DBD-P/T (1:3) (d) and DBD-T(e); Figure S5: Mott-Schottky plots of DBD-P (a), DBD-P/T (3:1) (b), DBD-P/T (1:1) (c), DBD-P/T (1:3) (d) and DBD-T (e) at different frequencies in an aqueous solution of Na_2_SO_4_ (0.5 M); Table S1: Fitted decay time of these polymers; Table S2: The specific parameters of the main component in the equivalent circuit diagram; Table S3: Selective oxidation of benzylamine with different dosages of DBD-T; Table S4: Photocatalytic methyl p-tolyl sulfide oxidation experiments by DBD-T; Table S5: The blue light-mediated oxidation of substrates on DBD-T with O_2_ to the corresponding products; Table S6: The photocatalytic aerobic oxidation of sulfides with oxygen over DBD-T driven by blue light; Table S7: Photocatalytic oxidative coupling of benzylamine.

## Abbreviations

The following abbreviations are used in this manuscript:

D-A	donor–acceptor
CPPs	conjugated porous polymers
POPs	porous organic polymers
CMPs	conjugated microporous polymer
ROSs	reactive oxygen species
BTDO	dibenzothiophene-S, S-dioxide
DBD	4,8-bis(thiophen-2-yl) benzo [1,2-b:4,5-b’] dithiophene
LUMO	lowest unoccupied molecular orbital
ACN	acetonitrile
MeOH	methanol
