# Supplementary material for: Thiophene–Sulfone-Based D-A Conjugated Porous Polymers: Acceptor Regulation for Efficient Blue Light-Driven Selective Aerobic Oxidation of Sulfides and Amines
Source: Molecules. 2026 Mar 24;31(7):1065. doi: 10.3390/molecules31071065 (PMC13075132; doi:10.3390/molecules31071065)

Supporting Information

Thiophene–Sulfone-Based D-A Conjugated Porous Polymers: Acceptor Regulation for Efficient Blue-Light-Driven Selective Aerobic Oxidation of Sulfides and Amines

Ruiyao Li [1], Fei Zhao [1, 2*], Qun Li [1], Shuai Feng [1], Chang-An Wang [1], Yinfeng Han [1,*], Xueli Cheng [1] and Jinsheng Zhao [3,*]

[1] College of Chemistry and Chemical Engineering, Taishan University, Taian 271000, China; liry2026@163.com (R. L.); liqun75@tsu.edu.cn (Q. L.); shuaifeng@tsu.edu.cn (S. F.); zhaofei@tsu.edu.cn (F. Z.); han@tsu.edu.cn (Y. H.); wangcha@tsu.edu.cn (C. W.); x_cheng@tsu.edu.cn (X. C.)
[2] College of Chemistry and Chemical Engineering, Qufu Normal University, Qufu 273165, China; zhaofei@tsu.edu.cn (F. Z.)
[3] College of Chemistry and Chemical Engineering, Liaocheng University, Liaocheng 252059, China; j.s.zhao@163.com (J. Z.)
* Correspondence: zhaofei@tsu.edu.cn (F. Z.); j.s.zhao@163.com (J. Z.); han@tsu.edu.cn (Y. H.)

Section S1. General information

1.1 Characterizations instrumentations

The thermogravimetric analysis (TGA) of the photocatalysts was conducted on a Netzsch STA449C TG/DSC thermal analyser and accompanied by a nitrogen atmosphere in the temperature range from 20 °C to 800 °C. Fourier Transform infrared (FT-IR) spectra (Nicolet iS50） was used to get the spectrum of the polymers in the range of 500-4000 cm-1. Powder X-ray diffraction (PXRD) measurements were carried out on a RigakuD/max2500 X-ray forward diffractometer with diffraction angles selected from 3° to 80°, using Cu-Ka radiation and scanning steps of 0.02 for powder x-ray diffraction (PXRD). The UV-Vis absorption of the samples was determined using a Carry 5000 UV-Vis-NIR spectrophotometer (Varian) in the wavelength range of 200 nm to 800 nm with $BaSO_4$ as a reference. The surface areas and pore size distributions were measured by Nitrogen isotherm adsorption-desorption at 77 K using ASAP 2460 (Micromeritics) volumetric adsorption analyzer. The positions of the valence bands of the samples and the valence states of the contained elements were analysed using X-ray photoelectron spectroscopy (XPS, scalab250Xi). Substrate conversion and selectivity were measured using ^{1}H NMR spectroscopy (Advanced NEO 500 MHz Spectrometer, Bruker BioSpin International AG, Switzerland). The morphology of the photocatalysts was measured using scanning electron microscopy (SEM) (Thermo Fisher Scientific FIB-SEM GX4) and transmission electron microscopy (TEM) (Jem-2100F Jeol). Both photoluminescence spectroscopy (PL) and time-resolved fluorescence spectroscopy (TRPL) were used to acquire the data using a

fluorescence spectrometer (FLS1000). The Electron paramagnetic resonance spectroscopy (EPR) was measured using a China instru&Quantumtech (Hefei) EPR200-Plus with continues-wave X band frequency.

1.2 Chemicals

2,7-dibromopyrene, 2,2'-((2,6-bis(4,4,5,5-tetramethyl-1,3,2-dioxaborolan-2-yl)benzo[1,2-b:4,5-b']dithiophene-4,8-diyl)bis(thiophene-5,2-diyl))bis(4,4,5,5-tetramethyl-1,3,2-dioxaborolane), tetrakis(triphenylphosphine)palladium(0) ($Pd(PPh_3)_4$) 2-aminopyridine (98%), 2-thiophenemethylamine (98%) and 4-tert-Butylbenzylamine (98%) were obtained from Zhengzhou Alfachem Co., Ltd. 2,2,6,6-tetramethyl-L-piperidine-N-oxyl (TEMPO, 98%), 5,5-dimethyl-1-pyrroline N-oxide (DMPO, 98%), and tri-o-tolylphosphine (P(o-tol)3, 98%) were purchased from J&K Scientific. N,N-dimethylformamide (DMF), Benzylamine (99%), 4-chlorobenzylamine (98%), 2-methoxybenzylamine (97%), 4-bromobenzylamine (96%), 4-fluorobenzylamine (99%), 4-methoxybenzylamine (99%), 2-bromobenzylamine (99%), 4-methylbenzylamine (98%), 4-chlorothioanisole (98%), methyl p-tolyl sulfide (99%), methyl p-tolyl sulfide (98%), 4-bromothioanisole (98%), 4-methoxythioanisole (98%), dimethyl sulfoxide (DMSO, AR, >99%), chloroform-d ($CDCl_3$, 99.8%), acetonitrile (MeCN, AR, >99%), 1,4-benzoquinone (p-BQ, 99%), triethanolamine (TEA, AR), tert-butyl alcohol (t-BuOH, AR, >99%) benzotrifluoride (99%) were bought from Aladdin Co., Ltd. Tetrahydrofuran (THF, AR, >99.0 %) purchased from Xilong Chemical Co., Ltd. Hydroquinone （HQ， AR， >99.0 %）was purchased from Shanghai McLean Biochemical Technology Co., Ltd. Ethyl acetate (EAC, AR, >99.5 %), trichloromethane (AR), anhydrous ethanol (EtOH, AR, 99.7 %), anhydrous methanol (MeOH, AR, 99.5 %) were purchased from Yantai Far East Fine Chemical Co., Ltd. Silver nitrate ($AgNO_3$, 99.8%) was purchased from Shanghai Shiyi Chemistry Reagent Co.,Ltd.

1.3 Synthesis of polymers

1.3.1 Preparation of 3,7-dibromodibenzothiophene-S,S-dioxide

NBS (1.64 g, 9.24 mmol) was added in several portions to a concentrated sulfuric acid (30 mL) solution of dibenzodithiophene (1 g, 4.62 mmol). The resulting mixture was carefully poured into an ice/water mixture and carefully stirred at 0°C for 24 hours. The off-white solid was washed with 20% aqueous sodium bicarbonate, water and then dried to obtain a white solid. The product was further

recrystallized from chloroform to give white crystals in 60% yield. The successful synthesis of these monomers was confirmed as shown in the NMR spectra of Figure S1.

^{1}H NMR (500 MHz, Chloroform-d) δ: 7.94 (d, J = 1.5 Hz, 2H), 7.78 (d, J = 1.8 Hz, 2H), 7.65 (d, J = 8.2 Hz, 2H).

^{13}C NMR (126 MHz, Chloroform-d) δ: 138.90, 137.16, 129.61, 125.61, 124.61, 122.92.

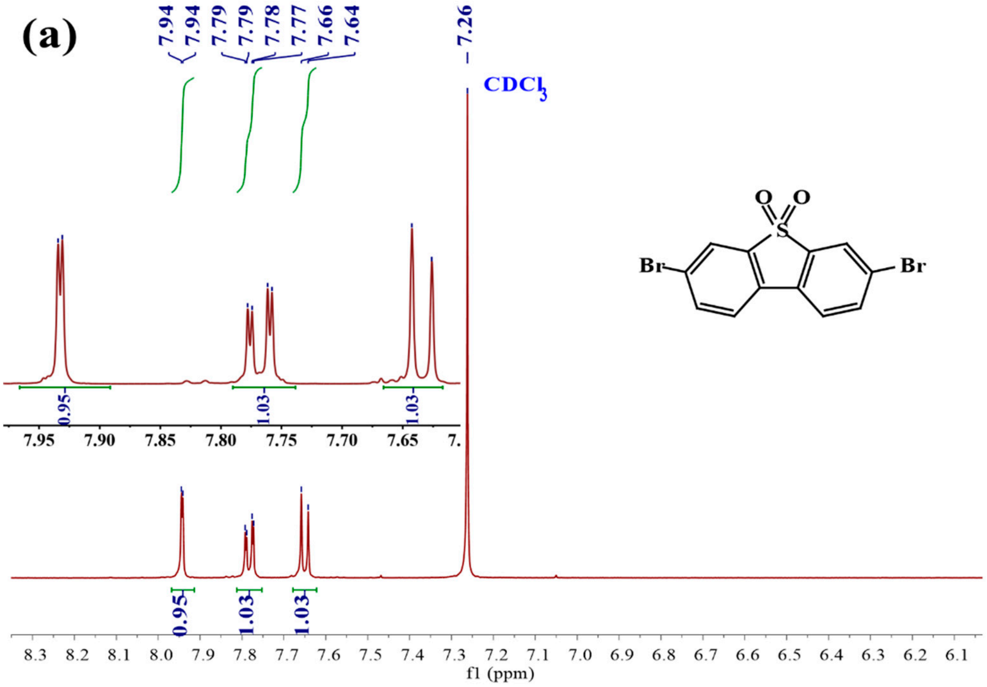

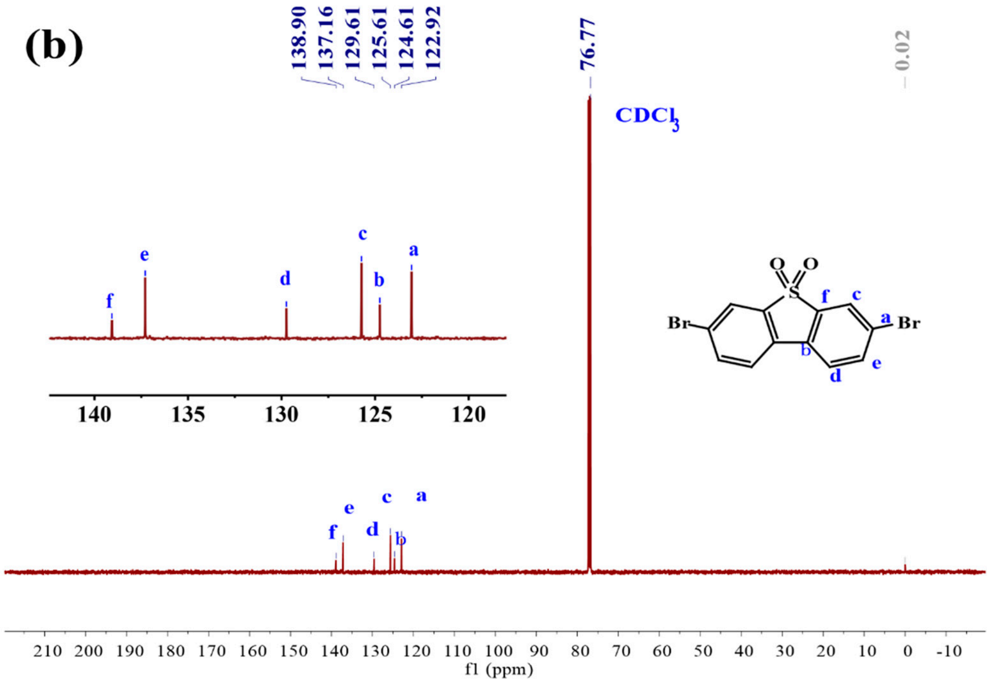

Figure S1 ^{1}H NMR (a) and ^{13}C NMR (b) spectrum of 3,7-dibromodibenzothiophene-S,S-dioxide.

1.3.2 Preparation of polymers

1.3.2.1 Preparation of DBD-P

The polymer DBD-P was synthesized as follows: M1 (699.1 μmol, 251.7 mg) and M2 (331.4 μmol, 300.0 mg) were dispersed into a flask containing an aqueous solution of potassium carbonate (2 M, 12.0 mL) and a solution of DMF (30.0 mL). Subsequently, the reaction flask was evacuated and backfilled three times with nitrogen. Pd $(PPh_3)_4$ (30.0 μmol, 35 mg) was then added to the flask under N_2 atmosphere. The mixture was heated and stirred at 120 °C for 72 h under nitrogen as a protective atmosphere. At the end of the reaction, it was filtered through reduced pressure and washed with deionized water, methanol and chloroform. The product was dried under vacuum at 60 °C for 12 hours. After a typical experimental procedure, a purplish-red solid was finally obtained. Product quality: 320 mg; yield: 79.22%.

1.3.2.2 Preparation of DBD-P/T (3:1)

The polymer DBD-P/T (3:1) was synthesized as follows: dispersion of M1 (524.4 μmol, 188.8 mg), M2 (331.4 μmol, 300.0 mg), and M3 (174.8 μmol, 65.4 mg) into flasks containing an aqueous solution of potassium carbonate (2 M, 12.0 mL) and a solution of DMF (30.0 mL). Subsequently, the reaction flask was evacuated and backfilled three times with nitrogen. Pd $(PPh_3)_4$ (30.0 μmol, 35 mg) was then added to the flask under N_2 atmosphere. The mixture was heated and stirred at 120 °C for 72 h under nitrogen as a protective atmosphere. At the end of the reaction, it was filtered through reduced pressure and washed with deionized water, methanol and chloroform. The product was dried under vacuum at 60 °C for 12 hours. After a typical experimental procedure, an orange-brown solid was finally obtained. Product quality: 267.6 mg; Yield: 65.84%.

1.3.2.3 Preparation of DBD-P/T (1:1)

The polymer DBD-P/T (1:1) was synthesized as follows: dispersion of M1 (357.8 μmol, 128.8 mg), M2 (331.4 μmol, 300.0 mg), and M3 (349.7 μmol, 130.8 mg) into flasks containing an aqueous solution of potassium carbonate (2 M, 12.0 mL) and a solution of DMF (30.0 mL). Subsequently, the reaction flask was evacuated and backfilled three times with nitrogen. Pd $(PPh_3)_4$ (30.0 μmol, 35 mg) was then added to the flask under N_2 atmosphere. The mixture was heated and stirred at 120 °C for 72 h under nitrogen as a protective atmosphere. At the end of the reaction, it was filtered through reduced pressure and washed with deionized water, methanol and chloroform. The product was dried under vacuum at 60 °C for 12 hours. After an operation not identical to 2.2.1, a red solid was finally obtained.

Product quality: 249 mg; yield: 60.46%.

1.3.2.4 Preparation of DBD-P/T (1:3)

The polymer DBD-P/T (1:3) was synthesized as follows: dispersion of M1 (194.4 μmol, 70.0 mg), M2 (331.4 μmol, 300.0 mg), and M3 (524.33 μmol, 196.1 mg) into flasks containing an aqueous solution of potassium carbonate (2 M, 12.0 mL) and a solution of DMF (30.0 mL). Subsequently, the reaction flask was evacuated and backfilled three times with nitrogen. Pd $(PPh_3)_4$ (30.0 μmol, 35 mg) was then added to the flask under N_2 atmosphere. The mixture was heated and stirred at 120 °C for 72 h under nitrogen as a protective atmosphere. At the end of the reaction, it was filtered through reduced pressure and washed with deionized water, methanol and chloroform. The product was dried under vacuum at 60 °C for 12 hours. After a typical experimental procedure, a reddish brown solid was finally obtained. Product quality: 301.6 mg; yield: 72.09%.

1.4 Electrochemical measurements [1-3]

Electrochemical impedance spectra (EIS) was executed on a CHI660E (Chenhua, Shanghai) electrochemical workstation using a standard three-electrode system.Firstly, the catalyst slurry was prepared by adding 10 mg of photocatalyst, 1 ml of isopropanol and 3 μL of naphthol into a ball milling jar for 4.5 h in order to obtain a well-mixed catalyst slurry that was ultrasonicated before use. Next, 10 μL of catalyst slurry was pipetted onto the platinum plate electrode (Φ 3 mm) using a pipette gun and dried under an infrared lamp to produce a catalyst-modified platinum plate electrodeas the working electrode, with the Ag/AgCl electrode and platinum flake as the reference electrode and counter electrode, respectively. Finally, the working electrode was calibrated using cyclic voltammetry with a mixture of $KCl/K_3Fe(CN)_6$ as the electrolyte before EIS measurements. Subsequently, an aqueous sodium sulphate solution (0.5 M) was used as the electrolyte and Electrochemical impedance tests were performed in the frequency range of 0.01 Hz to 100 k Hz.

1.5 Transient photocurrent measurements

The transient photocurrent response (I-t) was also measured on a CHI660E (Chenhua, Shanghai) electrochemical workstation, also using a standard three-electrode system. In the three-electrode system Pt sheet (1 cm × 1 cm was used as counter electrode, Ag/AgCl electrode was used as reference electrode, and 0.5 M aqueous sodium sulfate solution was used as electrolyte, which differed from the electrochemical impedance spectroscopy (EIS) determination in that the working electrode was a catalyst-modified indium tin oxide (ITO) electrode. Catalyst slurry was prepared by adding 10 mg of

catalyst to a mixture of 1 mL of isopropanol and 30 μL of Nafion (5%), which was completely dispersed in an ultrasonic cleaner for 30 min before use. To prepare ITO electrodes, 20 μL of the above polymer slurry was coated on ITO/glass electrodes with a surface area of 1 cm × 1 cm and irradiated under an infrared lamp aiming to dry the catalyst slurry. During the measurements, a 300 W Xe lamp was used as the light source, and the light switch was switched on and off for one cycle of 20 s. The light was switched off and on for one cycle of 20 s.

1.6 Supplementary Equation

$$ahv = A\left(hv - E_{\mathrm{g}}\right)^{\frac{1}{2}} \quad \text{(S1)}$$

$$E_{VB}(vs. NHE) = \varphi + E_{VBM} - 4.5\ \mathrm{e}V \quad \text{(S2)}$$

$$E_{VB} = E_{CB} + E_g \quad \text{(S3)}$$

$$I(t) = A_1\, exp(-t/\tau_1) + A_2\, exp(-t/\tau_2) \quad \text{(S4)}$$

In Equation S1, α, h, v, A, and E_{g} are the absorption coefficient, Planck constant, light frequency, a constant, and the band gap respectively.

In Equation S2, E_{VB}, E_{VBM} , φ are the valence band, the valence band maximum positions, the work function of the test instrument, φ = 4.47 eV (*vs.* vacuum) in this work.

In Equation S3, E_{CB} is the conduction band.

In Equation S4, A_1 and A_2 represent the amplitudes, and τ_1 and τ_2 can be defined as the corresponding emission lifetimes, respectively. t stands for time; I(t) represents the fluorescence intensity when time is t.

1.7 Quantitative Nuclear Magnetic Resonance (q-NMR)

The q-NMR enables the accurate quantification of particular compounds within a sample. For the reliability and consistency of quantitative NMR assays, it is essential to follow defined conditions and step.

Step 1: Choosing a Suitable Internal Standard (such as toluene): Select a stable compound with a known concentration to serve as the internal standard. Ensure that its chemical shift does not coincide with that of the analyte and that it dissolves well and remains stable in the chosen solvent.

Step 2: Sample Preparation: Mix the sample to be tested with the internal standard at an appropriate ratio, and ensure that both are completely dissolved in the solvent ($CDCl_3$).

Step 3: Instrument Calibration: Calibrate the NMR instrument using standard samples or solutions with known concentrations to ensure that the integral intensity is proportional to the actual concentration.

Step 4. Data Acquisition: Collect the one-dimensional proton spectrum (^1H NMR) and ensure that all signals are within the linear range. Avoid signal saturation or clipping.

Step 5. Analyzing Data: Determine the concentration of the analyte by comparing the integration values of the analyte and the internal standard, using the following formula:

$$[c]_{analyte} = \frac{I_{analyte} \times [c]_{in} \times M_{in}}{I_{in} \times M_{analyte}}$$

$[c]_{analyte}$ the concentration of analyte

$I_{analyte}$ the integral strength of analyte

$[c]_{in}$ the concentration of internal standard

M_{in} the molar mass of internal standard

I_{in} the integral strength of internal standard

$M_{analyte}$ the molar mass of the analyte

Step 6. Repeatability and Precision: To ensure the reliability of the results, three parallel measurements will be undertaken and the mean values will be calculated.

Section S2. Results

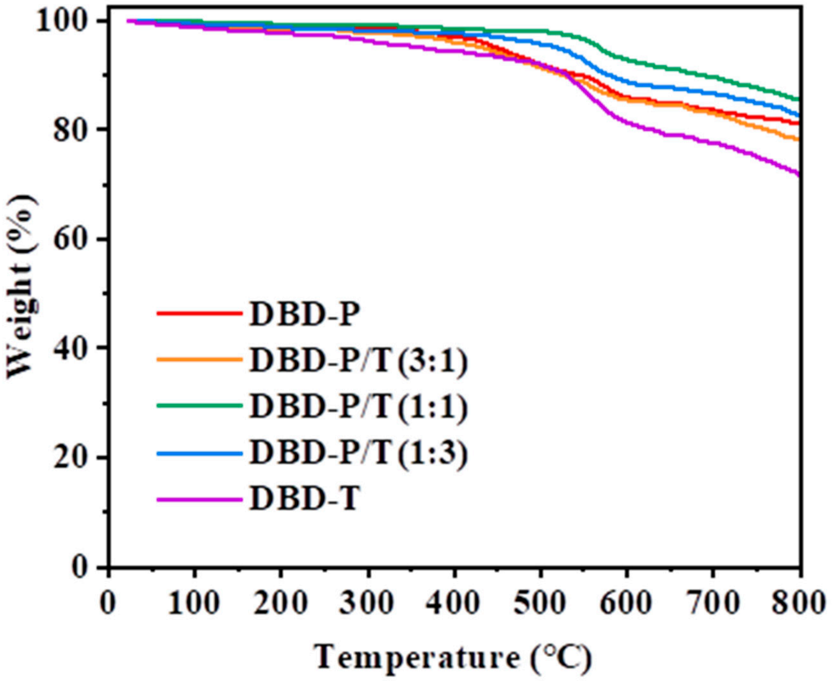

Figure S2. Thermogravimetric curves of polymers.

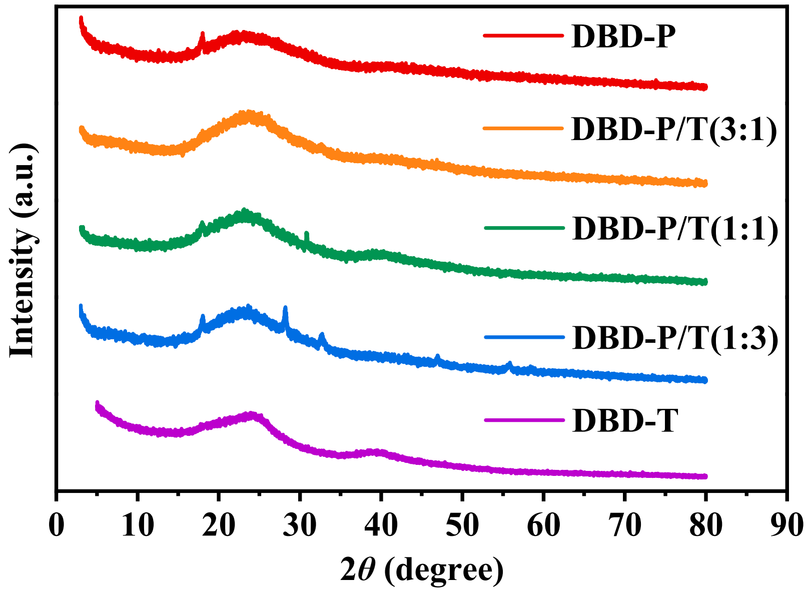

Figure S3. X-ray diffraction spectra of polymers.

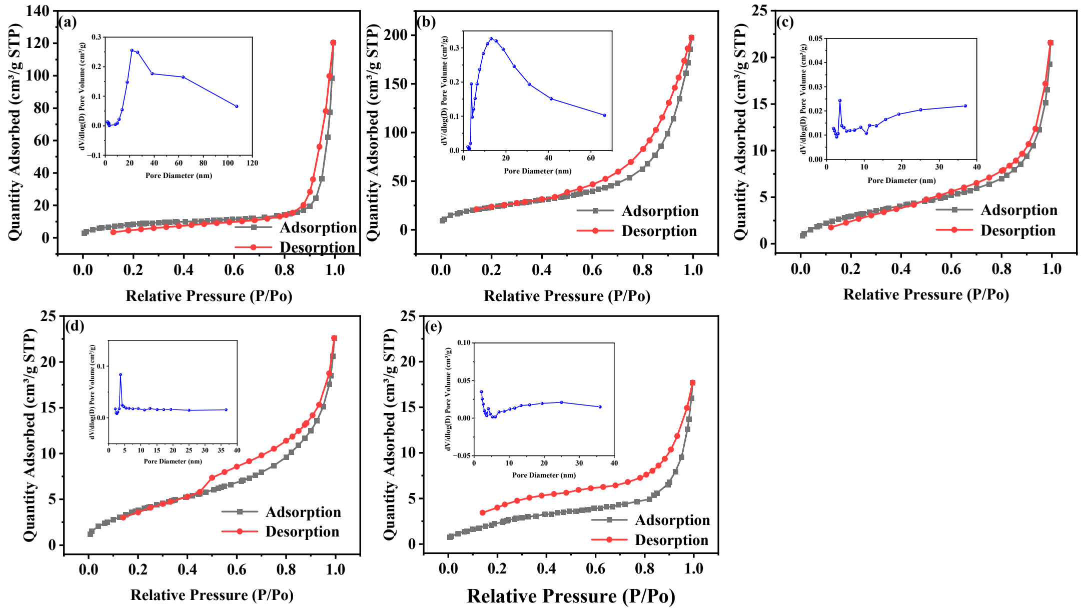

Figure S4. Nitrogen isothermal adsorption and desorption curves of DBD-P (a), DBD-P/T (3:1) (b), DBD-P/T (1:1) (c), DBD-P/T (1:3) (d) and DBD-T(e).

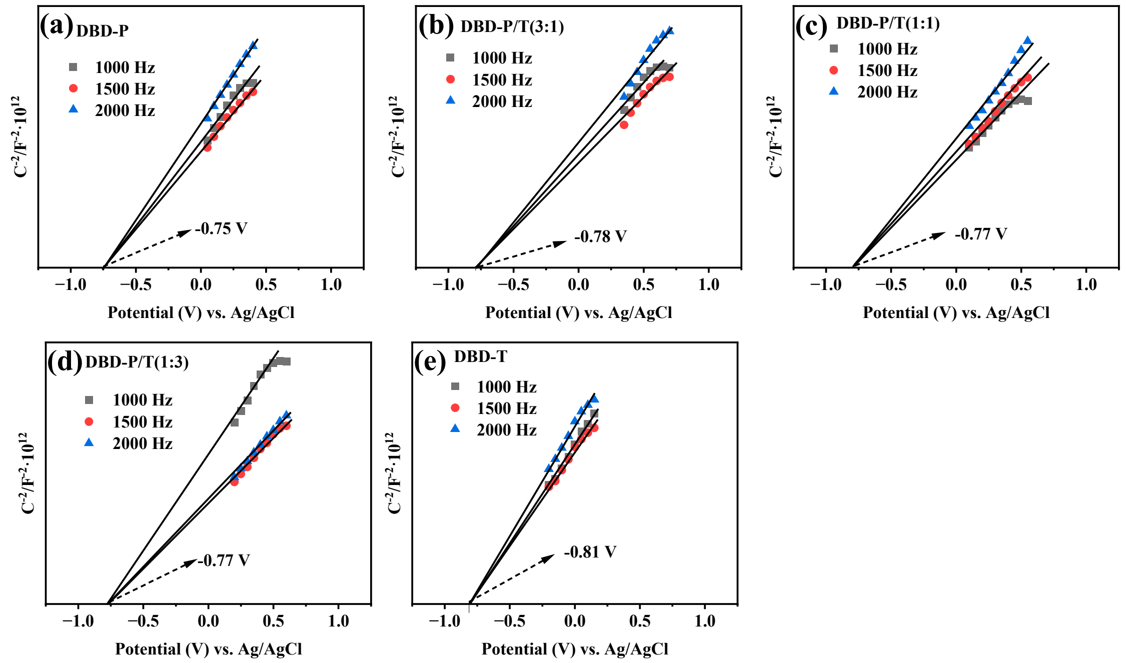

Figure S5. Mott-Schottky plots of DBD-P (a), DBD-P/T (3:1) (b), DBD-P/T (1:1) (c), DBD-P/T (1:3) (d) and DBD-T(e) at different frequency in an aqueous solution of Na_2SO_4 (0.5 M).

Table S1. Fitted decay time of these polymers.

polymer	τ_1(ns)	τ_2 (ns)	τ_{ave} (ns)
DBD-P	1.3691	1.3689	1.3690
DBD-P/T(3:1)	1.7464	1.7462	1.7463
DBD-P/T(1:1)	1.8233	1.8235	1.8234
DBD-P/T(1:3)	2.0606	2.0605	2.0605
DBD-T	2.3661	2.3662	2.3661

Table S2. The specific parameters of the main component in the equivalent circuit diagram.

**Component**	**Unit**	**DBD-P**	**DBD-P/T (3:1)**	**DBD-P/T (1:1)**	**DBD-P/T (1:3)**	**DBD-T**
Solution Resistance (R_s)	Ω	89.52	23.13	24.77	22.69	38.42
Charge Transfer Resistance (R_p)	kΩ	15.12	10.53	9.03	8.94	8.67

Table S3. Selective oxidation of benzylamine with different dosages of DBD-T.[a]

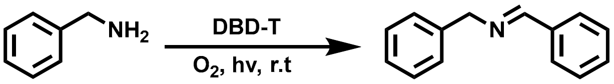

**Catalyst quantity (mg)**	**Conv. (%)**	**Sel. (%)**
**2**	**97**	**99**
**3**	**91**	**99**
**4**	**91**	**99**
**5**	**74**	**98**

[a] Reaction conditions: catalyst (DBD-T), benzylamine (0.3 mmol), ACN (1 mL), 0.5h, O_2, r.t, blue LEDs.

Table S4. Photocatalytic methyl p-tolyl sulfide oxidation experiments by DBD-T.[a]

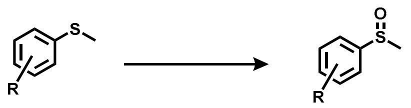

Entry	Catalyst	*hv*	O_2	Scavenger	Conv. (%)	Sel. (%)
**1**	+	+	+	**-**	**96**	**99**
**2**	-	+	+	**-**	**Not detected**	**-**
**3**	+	-	+	**-**	**Not detected**	**-**
**4**	+	+	N_2	**-**	**Not detected**	**-**
**5**	+	+	**Air**	**-**	**31**	**91**
**6**	+	+	+	**t-BuOH**	**90**	**97**
**7**	+	+	+	**TEOA**	**57**	>99
**8**	+	+	+	***p*-BQ**	**3**	>99
**9**	+	+	+	**$AgNO_3$**	**57**	**97**
**10**	+	+	+	**HQ**	**21**	>99
**11**	+	+	+	**TEMPO**	**24**	>99
**12**[b]	+	+	+	**-**	**2**	>99

[a] Reaction conditions: substrate (0.3 mmol), Photocatalyst (2.0 mg), MeOH (1.0 mL), r.t, 0.5h, blue LEDs.

[b]ACN as the solvent.

Table S5. The blue light-mediated oxidation of substrates on DBD-T with O_2 to the corresponding products. [a]

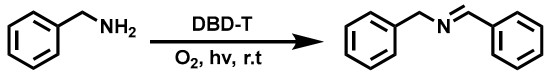

Entry	Substrate	Product	Conv. (%) [b]	Sel (%) [b]
1	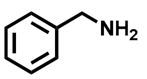	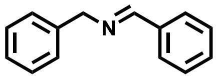	98	99
2	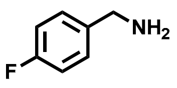	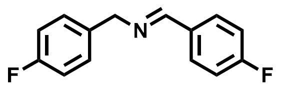	92	99
3	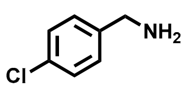	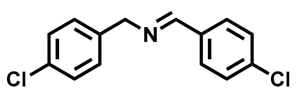	81	99
4	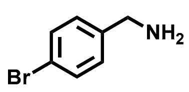	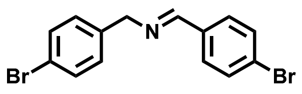	79	99

5	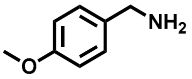	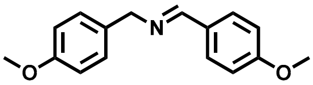	99	99
6	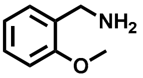	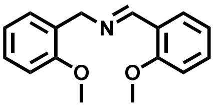	80	99
7	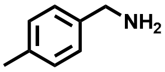	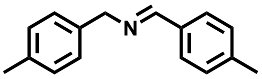	98	99
8	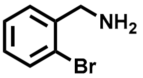	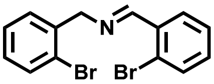	74	99
9	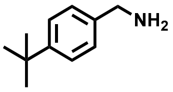	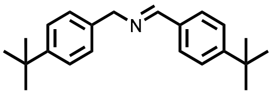	85	99
10	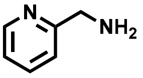	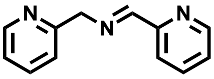	91	99
11	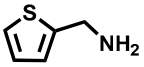	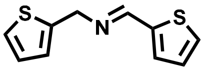	76	99

[a] Reaction conditions: substrate (0.3 mmol), Photocatalyst (2.0 mg), ACN (1.0 mL), blue LEDs, r.t, O_2 (1 atm), 0.9 h.
[b] Conversion and selectivity of the substrates were determined by ^{1}H-NMR.

Table S6. The photocatalytic aerobic oxidation of sulfides with oxygen over DBD-T driven by blue light. [a]

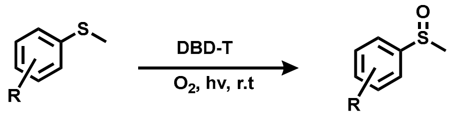

Entry	Substrate	Product	Conv. (%) [b]	Sel (%) [b]
1	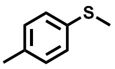	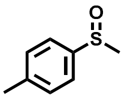	98	99
2	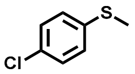	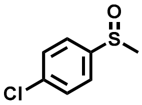	82	95
3	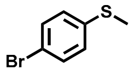	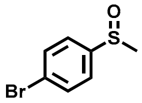	57	92
4	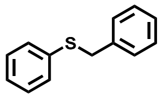	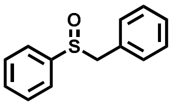	75	96

5	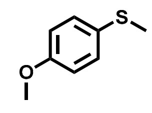	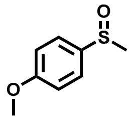	99	96

[a] Reaction conditions: substrate (0.3 mmol), Photocatalyst (2.0 mg), MeOH (1.0 mL), blue LEDs, r.t, O_2 (1 atm), 0.9h.
[b] Conversion and selectivity of the substrates were determined by ^{1}H-NMR.

Table S7. Photocatalytic oxidative coupling of benzylamine.[a]

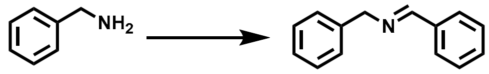

Entry	Catalyst	*hv*	O_2	Conv. (%)	Sel. (%)
**1**	+	+	+	**97**	**99**
**2**	-	+	+	**Not detected**	-
**3**	+	-	+	**Not detected**	-
**4**	+	+	N_2	**Not detected**	-
**5**	+	+	**Air**	**74**	**98**

[a] Reaction conditions: substrate (0.3 mmol), Photocatalyst (2.0 mg), ACN (1.0 mL), r.t, 0.5h, blue LEDs.

Section S3. ^{1}H NMR, ^{13}C NMR, and HRMS of the Products.

(E)-N-benzyl-1-phenymethanimine

^{1}H NMR (500 MHz, Chloroform-d) δ 8.41 (s, 1H), 7.79-7.78 (d, 2H), 7.27-7.23 (m, 8H), 4.84 (s, 2H).

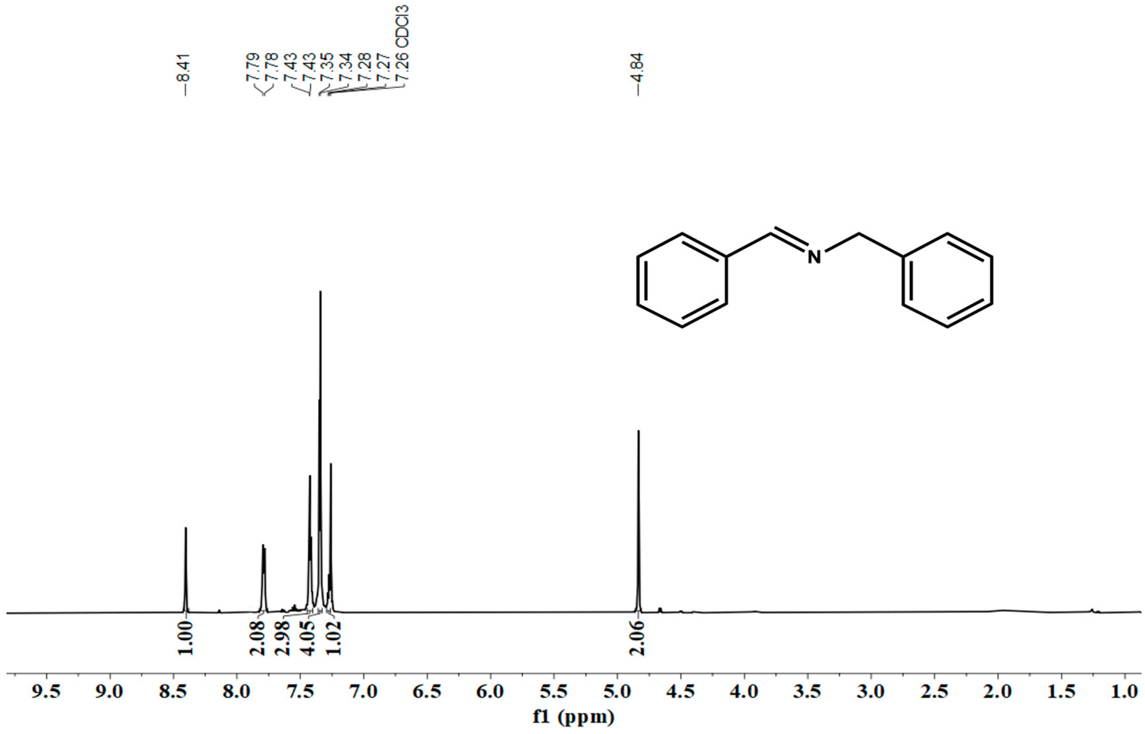

1-methyl-4-(methylsulfinyl)benzene.

^{1}H NMR (500 MHz, Chloroform-d) δ 7.54-7.53 (d, 2H),7.34-7.32 (d, 2H), 2.70 (s, 3H), 2.41 (s, 3H).

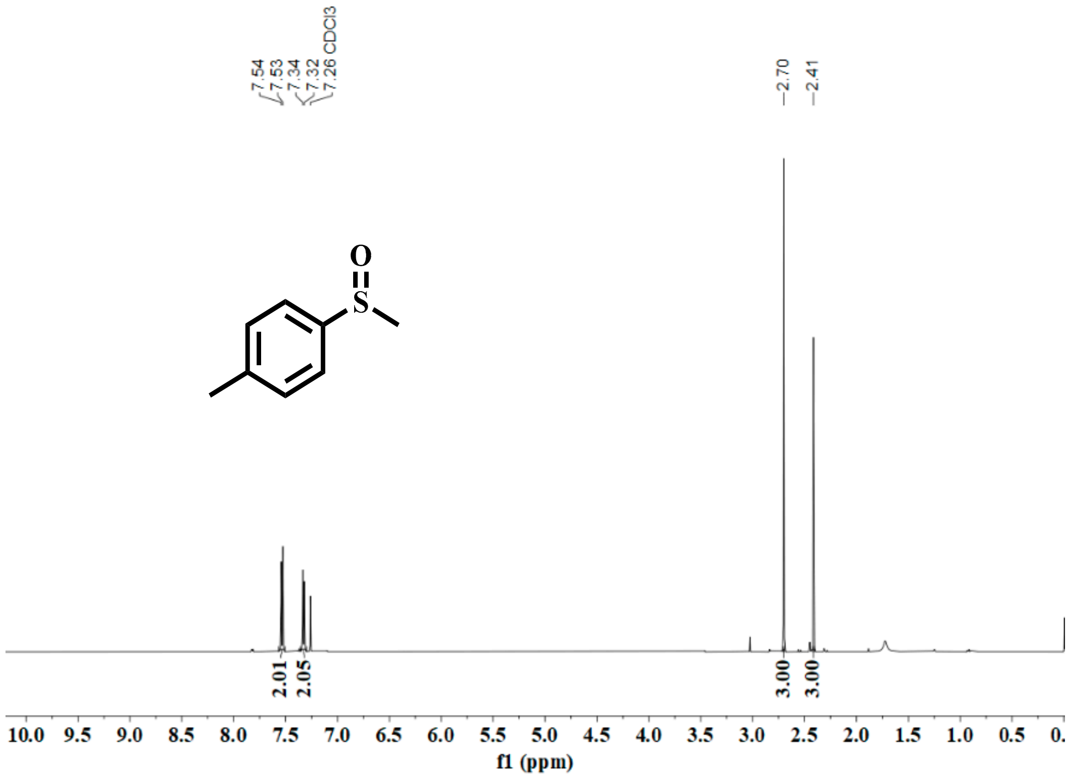

Supplement: Supplementary file 1 [file molecules-31-01065-s001.zip › molecules-4184792-supplementary.pdf]
